# Let's Not Miss the Treatable Ones: Two Cases of Neonatal Sepsis Due to Malaria

**DOI:** 10.7759/cureus.27731

**Published:** 2022-08-06

**Authors:** Madhavi Majety, Priyanka Majety, Venkataramana Kammili

**Affiliations:** 1 Pediatrics and Child Health, Anurag Hospital for Children, Vijayawada, IND; 2 Endocrinology, Diabetes and Metabolism, Beth Israel Deaconess Medical Center, Harvard Medical School, Boston, USA; 3 Neonatology, Nori Multispeciality Hospital, Vijayawada, IND

**Keywords:** neonatal malaria, artemisinin, congenital malaria, neonatal jaundice, neonatal sepsis

## Abstract

Congenital malaria is the direct infection of an infant with a malarial parasite from the mother either during pregnancy or at birth. Neonatal malaria occurs due to an infective mosquito bite after birth. Neonatal and congenital malaria (NCM) can occasionally present with life-threatening neonatal sepsis and rarely with neonatal jaundice. These conditions are typically managed by general pediatricians, especially in remote areas without access to specialized care. A high clinical index of suspicion is required to diagnose neonatal and congenital malaria, given that their presentation can mimic other more common neonatal conditions. We present two neonates with malaria, highlighting the importance of considering this treatable entity in the differential.

## Introduction

India contributes 70% of malaria cases and 69% of malaria deaths in the South-East Asia Region. Eighty-two percent of the country’s population lives in malaria transmission risk areas [1]. Despite India’s efforts to eliminate malaria, it remains a malaria high burden country [2]. Infection with malarial parasites during pregnancy is associated with poor birth outcomes, resulting in significant perinatal morbidity and neonatal mortality [3,4]. Congenital malaria is the direct infection of an infant due to transplacental transmission of the malarial parasite from the mother either in utero or during birth. Detection of asexual forms of malarial parasite in the peripheral smear of a newborn in the first seven days of life or later if there is no possibility of postpartum infection through infective mosquito bites confirms this diagnosis [5]. Neonatal malaria is vector-borne, caused by the bite of an infective mosquito after birth. Around 150 cases of congenital malaria are reported the world over [6,7].

We present two cases of malaria in neonates from a pediatric hospital with a level-2 neonatal unit from a tier two city in South India, highlighting the importance of considering an underdiagnosed condition which, if not entertained in the differential, can prove detrimental.

## Case presentation

Case 1

A term (38+1 weeks) female neonate, born to a primi mother and non-consanguineous parents by an uncomplicated vaginal delivery, presented at 18 hours of life with jaundice in November 2019. Mother had an uneventful pregnancy and labor. She was not on any medications, and there was no history of febrile illnesses during her pregnancy. Serum bilirubin of the baby was 18.4 mg/dl (exchange transfusion range- 18.5 mg/dl) at 18 hours of life. American academy of pediatrics nomograms was used in the management of hyperbilirubinemia. Intensive phototherapy started, and repeat serum bilirubin done at 24, 36, and 60 hours of life were 16 mg/dl (below exchange range), 13.5 mg/dl (above phototherapy range), and 10 mg/dl (below phototherapy range), respectively. Investigations revealed the mother’s and infant’s blood group to be B +ve. Direct Coombs test, which was done to rule out major and minor blood group incompatibilities, was negative, and a peripheral smear study showed a hemolytic picture with neutrophilia and a reticulocyte count of 33%, Glucose-6-phosphate dehydrogenase (G6PD) levels were normal. The osmotic fragility test was normal. Sepsis screening is done at admission, and 48 hours of life was negative. She was discharged on the fourth day of life and, on regular follow-up, was found to be thriving well.

On the 20^th^ day of life, the baby was brought with complaints of fever, rash, and inconsolable crying for one day. Systemic examination revealed a febrile infant with a macular rash all over the body. Investigations revealed a C - reactive protein (CRP) of 10 mg/dl, Erythrocyte sedimentation rate (ESR) of 52 mm/hour, Platelet count of 81,000/cumm, Hemoglobin (Hb) of 9.0 gm/dl and white blood cell count (WBC) of 5,400 cells/cumm. Complete urine examination (CUE) was normal. With a working diagnosis of probable late onset sepsis, the baby was admitted and started on empiric intravenous antibiotics after sending blood for culture. Lumbar puncture was discussed but not performed as per the family’s wishes. She continued to get intermittent spikes of fever, though feeding well. She eventually developed abdominal distension and sluggish bowel sounds. Repeat investigations after 48 hours showed a total leukocyte count (TLC) of 11,300/cumm, Hb of 7.8 gm/dl platelet, count of 45,000/cumm, and CRP of 35 mg/L.

**Figure 1:**
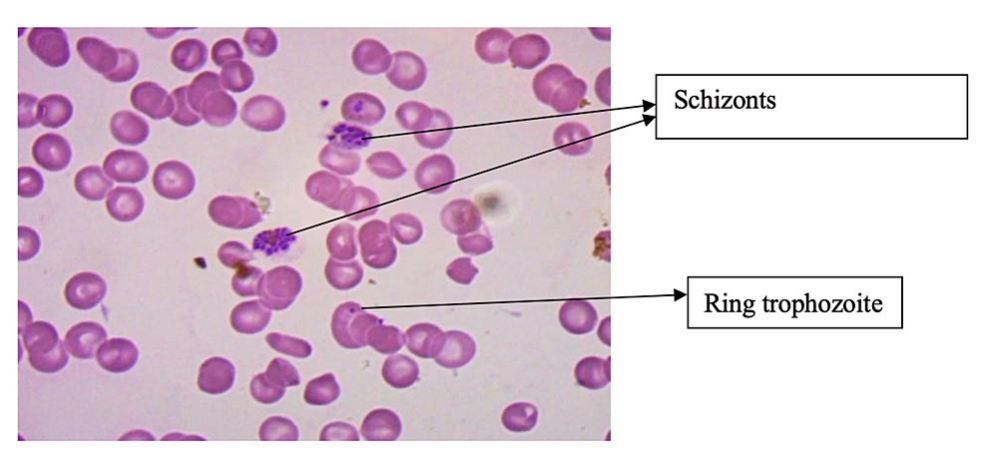
Peripheral smear showing schizonts and ring trophozoites of Plasmodium vivax

Abdominal ultrasound showed mild hepatomegaly and gallbladder wall edema. Chest and abdominal X-rays were normal. The cranial ultrasound was normal. Blood culture showed no growth. At this stage, a rapid test for malaria was done, which was found to be positive for Plasmodium vivax. Peripheral smear showed Schizonts and ring trophozoites of Plasmodium vivax (Figure 1).

She was treated with oral chloroquine in a dose of 10 mg/kg followed by 5 mg/kg after six hours, 24 hours, and 48 hours, according to the National Vector Borne Disease Control Program (NVBDCP) of India guidelines [8]. She became afebrile, and intravenous Antibiotics were stopped. The baby was discharged on oral iron and vitamin D3 supplements. The baby did not present for follow-up but, on telephonic inquiry, was found to be thriving well and gaining weight.

Case 2

A 26-day-old male newborn born to a fourth gravida mother presented with a two-day history of fever in March 2022. Her maternal history was notable for malarial fever in the first trimester and a history of blood transfusion in her third trimester due to anemia. The baby's birth history was unremarkable, with normal APGAR scores. Clinical examination on the day of presentation (26^th^ day of life) revealed a febrile neonate with pallor and hepatosplenomegaly.

Investigations revealed a WBC count of 10,500 cells/cumm (lymphocytes 51.4%, neutrophils 41%), Hb of 8 gm/dl, platelets of 77,000/cumm, CRP of 84 mg/L, Random blood sugar (RBS) of 96 mg/dl and normal CUE. COVID-19 testing was not performed. A rapid test for malaria was positive for both Plasmodium falciparum and Plasmodium vivax. A rapid test for Dengue was negative. A peripheral smear study of the mother was negative for the malarial parasite. The baby’s peripheral smear was also negative for malarial parasite but showed many fragmented red blood cells and target cells. Leukocytosis with a left shift of myeloid series of cells was present. G6PD levels were normal. The baby was admitted, and after sending blood for culture and sensitivity, was started on intravenous antibiotics and oral Artemether and Lumefantrine syrup, according to the NVBDCP guidelines. 

He became afebrile within 48 hours of starting oral Artemether and Lumefantrine syrup, following which antibiotics were stopped as blood culture showed no growth. Repeat investigations revealed a WBC count of 41,000/cumm, Hb of 9.9 gm/dl, platelets of 4,72000 /cumm, and C-reactive protein (CRP) came down to 4 mg/L. He was discharged on oral iron and vitamin D3 supplements. On further follow-up in the outpatient setting, he was gaining weight, and hepatosplenomegaly regressed.

## Discussion

Sepsis is one of the most common causes of death among neonates globally, especially in low and middle-income countries. The burden of neonatal sepsis in India is very high [9]. The most common cause of neonatal sepsis in India is a bacterial infection. There is also a significant burden of culture-negative sepsis leading to neonatal mortality [10]. Although not so uncommon, neonatal and congenital malaria (NCM) as a cause of neonatal sepsis is not frequently reported, presumably due to low suspicion among clinicians.

The most common presenting symptom of NCM is fever. Other features may include vomiting, diarrhea, anemia, jaundice, lethargy, poor feeding, respiratory distress, hepatosplenomegaly, convulsions, and sepsis-like presentation [11]. Although rapid tests are used for diagnosis, Giemsa-stained peripheral smear examination remains the gold standard despite its relative unavailability in remote areas and being prone to subjective errors. Plasmodium vivax is estimated to be the leading cause of NCM in Europe, while Plasmodium falciparum infection remains the commonest cause in Africa and India. While Chloroquine is still considered the drug of choice for Plasmodium vivax infections, due to the high prevalence of chloroquine resistance, Artemisinin-based combination therapy (ACT) is the recommended therapy for Plasmodium falciparum infections [8]. World Health Organization (WHO) currently recommends treatment with ACT for infants weighing less than 5 kg with Plasmodium falciparum malaria with the same dose as for children weighing 5 kg [11].

With these two case reports, we highlight the importance of considering malaria in the differential diagnosis of not only neonatal sepsis but also neonatal jaundice. In our first case, the neonate presented with jaundice and later with sepsis in the setting of congenital malaria. We could not find any other etiology for pathological jaundice. The first peripheral smear examination did not detect malarial parasites, which could have been due to low parasitemia. The possible reasons for low parasitemia could be due to the transplacental transfer of maternal antibodies (IgG) against malaria [12]. Fetal hemoglobin (HbF) may also confer some protection against high parasitemia [13]. Passive immunity may modify the severity of presentation as both IgG and HbF decrease with age, making the infants more susceptible. This may also delay the onset of symptoms up to three to six weeks after birth making it difficult to differentiate between the two entities of congenital and neonatal malaria. However, vector-born neonatal malaria can’t be ruled out. In the second case we presented, the maternal history was notable for malaria fever in the first trimester and blood transfusion in the third trimester. This presentation could also be probably due to congenital malaria with the delayed presentation though neonatal malaria cannot be ruled out. The smear study was negative for malarial parasites, though showed a hemolytic picture, which can be due to low parasitemia due to the aforementioned reasons. 

## Conclusions

Neonatal and congenital malaria is not as uncommon as previously thought, and a high index of suspicion is required. NCM can masquerade as hyperbilirubinemia and neonatal sepsis. Rapid tests and peripheral blood smear studies for malarial parasites should probably be routinely included in the workup of such neonates, which can pay rich dividends in helping clinicians to pick up this common and easily treatable but potentially fatal infection, especially in endemic countries like India.
